# Epirubicin-induced QT prolongation, monomorphic ventricular tachycardia, and response to beta blockade in long QT syndrome type 2

**DOI:** 10.1016/j.hrcr.2020.07.005

**Published:** 2020-07-14

**Authors:** Milos Kesek, Ylva Holmgren Stenlund, Steen M. Jensen

**Affiliations:** ∗ Department of Public Health and Clinical Medicine, Heart Centre, Umeå University, Umeå, Sweden; † Department of Radiation Sciences and Oncology, Umeå University, Umeå, Sweden

**Keywords:** Anthracycline, Beta-blocker, Cancer therapy, Cardio-oncology, Epirubicin, Long QT, LQT2, Metoprolol, Propranolol, QT interval prolongation

## Introduction

Congenital long QT syndrome (LQTS) is a group of cardiac channelopathies that are manifested through changes in repolarization. Patients are at increased risk of malignant ventricular arrhythmia, triggered by early afterdepolarization (EAD).^1^ The primary treatment consists of beta-blockade.^2^ The relative efficacy of various beta-blockers is a matter of debate and may vary among the genotypes.^3^, ^4^, ^5^ A survey of experts reported that most considered nonselective beta-blockers to be more effective. However, there is limited high-quality evidence and current guidelines abstain from opinion on this matter.^6^^,^^7^

**Key Teaching Points**

• Treatment of long QT syndrome (LQTS) patients with epirubicin may lead to a marked additional QT prolongation.
• Treatment with epirubicin in previously asymptomatic LQTS patients may lead to ventricular arrhythmias.
• Nonselective beta-blockers are often considered to be more effective than the selective ones in treatment of LQTS. This view is mainly based on expert opinion. The support in existing studies is weak and current guidelines offer no advice on the matter.
• The nonselective beta-blocker propranolol is more effective in suppressing these arrhythmias than the selective beta-blocker metoprolol.

Anthracyclines have been shown to prolong the QT interval (the time from the start of the Q wave to the end of the T wave on the electrocardiogram),^8^ but their arrhythmogenic potential in unselected population seems to be low.^9^ We located 1 case report of a patient with congenital LQTS (type 1) needing antineoplastic treatment with anthracyclines.^10^ Epirubicin is an anthracycline glycoside antineoplastic drug with cumulative cardiotoxic adverse effect. The drug is mainly eliminated through hepatic metabolism. The terminal half-life of the drug is estimated to be 16 hours, with large variations.^11^ This case report illustrates the consequence of exposing a patient with LQTS to epirubicin and describes a successful protection strategy against ventricular arrhythmia by propranolol where metoprolol proved to be less effective.

## Case report

A 40-year-old woman, weighing 74 kg, was identified on cascade screening to have LQTS type 2, with the mutation NM_000238.3(KCNH2):c.2593-2A>G, predicted to cause a splice-site disruption resulting in a truncated protein. This rare variant had not previously been reported in patients with LQTS or in healthy controls. It has been classified locally as pathogenic according to the guidelines of the American College of Medical Genetics. The proband was her younger sister, a previous multisport competitor who had an internal defibrillator implanted in 2005 after investigation of syncopal episodes during extreme physical strain and appropriate internal defibrillator shocks during follow-up. The family history contains cases of sudden death. A pedigree can be seen in the supplemental material.

The patient reported no prior symptomatic arrhythmia episodes. She has been treated with metoprolol slow-release 50 mg daily for more than 15 years. (In Sweden, metoprolol is generally available in slow-release form, as opposed to the nonselective beta-blockers.) At serial follow-up her QTc (QT interval corrected for heart rate) has been prolonged to approximately 480 ms. Over the years she has been physically active but not at a competitive level.

In 2019 the patient was diagnosed with ductal cancer in her left breast (T2(38 mm)pNX, luminal B, non-Her-2-amplified). Prior to surgery a decision was taken to treat her with neoadjuvant cytostatic chemotherapy including epirubicin 75 mg/m^2^ and cyclophosphamide 500 mg/m^2^ (EC75).

Prior to the first cytostatic pulse, a decision was made to increase her metoprolol dose to 75 mg daily. At this point her serum potassium concentration was normal. On day 1 of chemotherapy continuous telemetry monitoring was started and epirubicin 134 mg was administered intravenously over 2 hours, followed by cyclophosphamide 890 mg intravenously infused over 1 hour. The commonly administered antiemetic drug ondansetron was excluded owing to its known impact on the QT interval. Instead, metoclopramide and aprepitant were given against nausea as well as an elevated steroid dose; betamethasone 8 mg ×2 orally on day 1, followed on days 2–5 by 4 mg, 3 mg, 2 mg, and 1 mg, respectively.

**Figure 1:**
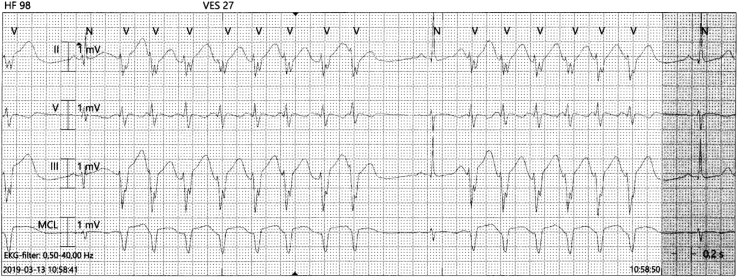
Example of the rhythm after start of the cytostatic pulse 1. Telemetry recording after the epirubicin infusion under treatment with metoprolol (paper speed 25 mm/s). The rhythm at this time consisted of runs of nonsustained monomorphic wide-QRS tachycardia with heart rate 125 beats per minute, separated by a few normal complexes. The patient complained about palpitations but was otherwise unaffected.

During the hours after start of the treatment the patient developed palpitations associated with frequent premature ventricular complexes and episodes of repetitive nonsustained monomorphic ventricular tachycardias up to 8 complexes (Figure 1). In periods, the rhythm consisted of the greater part of ectopic ventricular complexes separated by short periods of normal rhythm.

**Figure 2:**
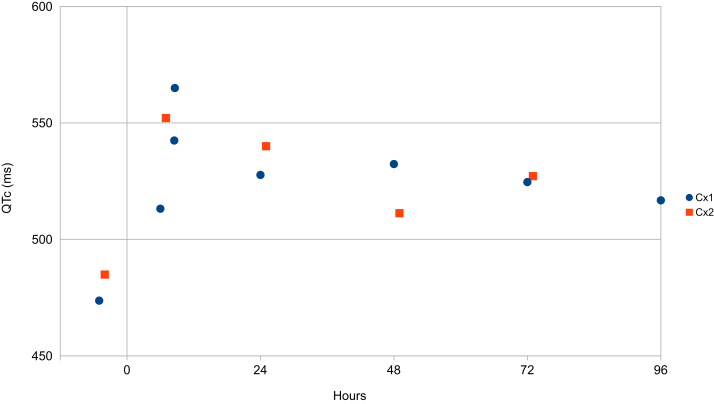
QTc during the cytostatic pulses 1 and 2. QTc during cytostatic therapy under treatment with metoprolol (Cx1) and propranolol (Cx2). The therapies Cx1 and Cx2 were otherwise identical. QTc was measured in a blinded random sequence of the electrocardiograms by an experienced operator. Bazett’s formula was used for heart rate correction. Epirubicin infusion was started at hour 0. The subsequent QTc prolongation was similar during both epirubicin pulses.

**Figure 3:**
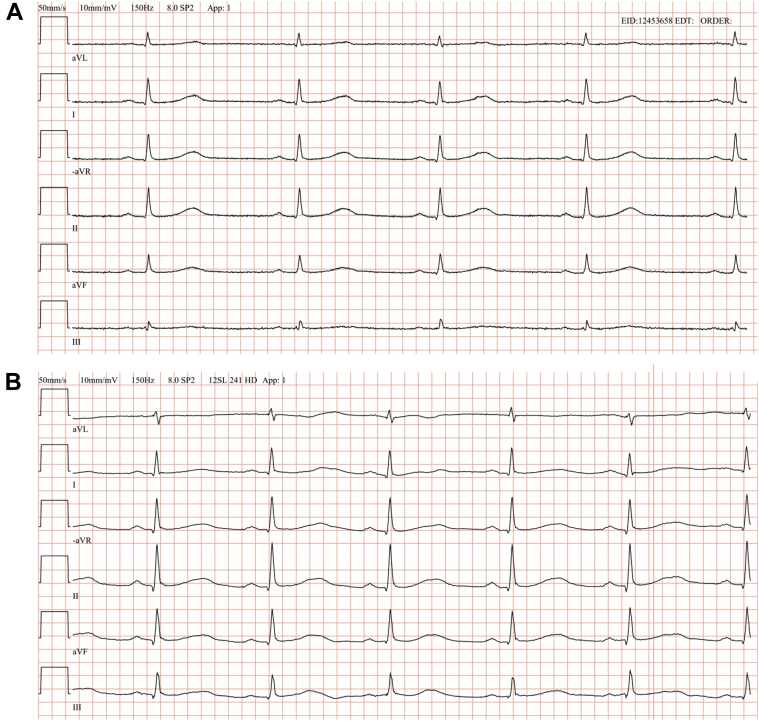
Electrocardiogram at baseline and at maximal QT interval (Cx1, day 1). Only extremity leads are shown (paper speed 50 mm/s). QT interval was 490 ms (**A**, baseline) and 530 ms (**B**, 8 hours after administration of epirubicin), respectively. QTc was 474 ms and 565 ms, respectively.

The arrhythmias decreased but re-emerged. The metoprolol dose was further increased to 100 mg daily. Potassium and magnesium were administered intravenously (40 mmol and 20 mmol, respectively). After 24 hours, the arrhythmias decreased. A high-sensitivity cardiac troponin T test was normal. During days 2 and 3, singular and coupled ventricular complexes were observed in the telemetry in decreasing frequency. On day 4 only singular ventricular complexes were noted and the patient was discharged. The mean heart rate during the entire period was 64 (± 7) beats per minute (bpm). QTc (corrected by Bazett’s formula) was 474 ms prior to the treatment, increased during day 1, and decreased slowly thereafter (Figures 2 and 3).

The second EC75 treatment, with the same composition as the first, was scheduled 3 weeks later. One week prior to the second treatment, metoprolol was switched to propranolol slow-release 160 mg daily, made available after application to the Swedish drug regulatory agency. The cytostatic treatment was administered and the patient was monitored by continuous telemetry over 3 days. No premature ventricular complexes or ventricular arrhythmias were observed during or after the second treatment. During this period the mean heart rate was 67 (± 6) bpm. The increase in QTc was comparable to that during the first treatment (Figure 2).

At this stage a magnetic resonance image showed no change in tumor size. The course of the neoadjuvant therapy was interrupted. The treatment with propranolol slow-release was continued and the patient underwent surgical resection, followed by adjuvant treatment with 4 pulses of docetaxel 80 mg/m^2^ under telemetry monitoring. No arrhythmias were noted. A proBNP test was normal. Subsequently the treatment was switched back from propranolol to metoprolol, according to the patient’s own wish. One year after the diagnosis the patient is doing well. Latest QTc was 496 ms, no clinical arrhythmias have occurred, and a 24-hour Holter recording 8 months after the first cytostatic treatment showed no premature ventricular complexes.

## Discussion

A marked additional QT prolongation was observed. Because of the risk associated with LQTS, the epirubicin dose for this patient was reduced from the conventional 90 to 75 mg/m^2^. Despite the reduction and simultaneous treatment with metoprolol slow-release, the administration of epirubicin on the first occasion was associated with repeated symptomatic ventricular arrhythmias. Switching to propranolol prophylaxis (also in slow-release form) prior to next cytostatic pulse with same composition was associated with a complete abolition of the ventricular arrhythmias during the treatment. This effect was not related to a change in the mean heart rate. The additional QT prolongation after epirubicin was similar during treatment with both beta-blockers. Metoclopramide is known to affect the QT interval and may have contributed to the marked QT prolongation. Metoclopramide was given on both treatment occasions and should therefore not have been involved in the arrhythmia that was observed during the first treatment only.

The patient was given betamethasone simultaneously with EC75 and in decreased dose during subsequent 4 days. Corticosteroids can decrease a prolonged QT interval by shortening the cardiac action potential and inhibit arrhythmias induced by prolonged QT.^12^ This should, however, not have affected the arrhythmia, since an identical steroid scheme was administered on both treatment occasions with EC75.

The typical arrhythmia in untreated LQTS consists of torsades de pointes, a rapid polymorphic ventricular tachycardia with characteristic oscillation of the electrical axis. This arrhythmia is thought to be induced by EAD.^1^ The arrhythmia seen during the initial hours after first epirubicin treatment, however, consisted of episodes with frequent, repetitive runs of 5–8 complexes of monomorphic ventricular tachycardia with heart rate 125–150 bpm.

A point of discussion is the extent to which the arrhythmia was related to the pre-existing LQTS. Anthracyclines are a common component of cytostatic therapy. They have been described to be associated with rare cases of sudden death, mostly late after the treatments, but few descriptions exist of ventricular arrhythmias immediately following the administration of anthracyclines. Reports mention ventricular premature beats. In 1 report of 29 patients with some cardiovascular comorbidity 2 asymptomatic episodes of nonsustained ventricular tachycardia were noted in 24-hour Holter recordings after administration of doxorubicin.^13^ To the best of our knowledge, incessant monomorphic ventricular tachycardia has not been described before. LQTS type 2 is not in itself associated with monomorphic ventricular tachycardia. A pure coincidence of these 2 unusual conditions, however, seems unlikely. Possibly, metoprolol was partially effective by preventing the triggering of consecutive EADs

It can only be speculated regarding the superiority of propranolol in this setting. In addition to its nonselective beta-blocking property, propranolol blocks Na^+^ channel activity in a manner similar to the local aesthetic drug group, including mexiletine.^14^ This might have been an advantage. Mexiletine is an effective antiarrhythmic in LQTS3 patients and it has recently been shown to shorten QTc in LQTS2 patients.^15^

## Conclusions

Treatment of patients with LQTS type 2 with epirubicin may lead to marked additional QT prolongation and precautions have to be taken. In the present case propranolol was more effective against ventricular arrhythmia than metoprolol. The relative efficacy of propranolol did not depend on differences in the heart rate or the QTc prolongation.

## Appendix Supplementary data

Supplemental material The pedigree. The graph shows the pedigree of the affected family originating from north-western Sweden. Squares: men. Circles: women. Crossed symbols: deceased individuals. E+ and filled symbol depicts individuals positive for the mutation NM_000238.3(KCNH2):c.2593-2A>G. E- depicts individuals, shown to be negative for the mutation. Symbols with central dot depict obligate carriers. The patient is shown as a red symbol. Two known cases of death at young age (17 and 26 years) can be seen. Additional two cases of sudden death (47 and 54 years) are known to have occurred in the extended family outside the pedigree view. b.1947 E+ b.1948 b.1975 E+ b.1972 Eb.1919 b.1919 b.1949 b.1954 b.1881 b.1894 b.1922 b.1928 Eb.1934 b.1967 Eb.1965 Eb.1960 Eb.1933 E+ b.1913 b.1951 b.1979 Eb.1980 E+ b.1983 b.1985 d. 17y E+ b.1912 b.1945 Eb.1941 b.1951 E+ b.1957 Eb.1949 b.1979 E+ b.1982 Eb.1980 E+ b.1979 b.2009 Eb.1969 b.2009 E+ b.2006 b.1974 b.2011 E+ b.2012 E+ b.1984 b.2017 E+ b.2019 b.2019 Ed. 26y
