# Supplementary material for: Epirubicin-induced QT prolongation, monomorphic ventricular tachycardia, and response to beta blockade in long QT syndrome type 2
Source: HeartRhythm Case Rep. 2020 Jul 14;6(10):729–32. doi: 10.1016/j.hrcr.2020.07.005 (PMC7573386; doi:10.1016/j.hrcr.2020.07.005)
Supplement: Supplemental material — The pedigree. The graph shows the pedigree of the affected family originating from north-western Sweden. Squares: men. Circles: women. Crossed symbols: deceased individuals. E+ and filled symbol depicts individuals positive for the mutation NM_000238.3(KCNH2):c.2593-2A>G. E- depicts individuals, shown to be negative for the mutation. Symbols with central dot depict obligate carriers. The patient is shown as a red symbol. Two known cases of death at young age (17 and 26 years) can be seen. Additional two cases of sudden death (47 and 54 years) are known to have occurred in the extended family outside the pedigree view. b.1947 E+ b.1948 b.1975 E+ b.1972 Eb.1919 b.1919 b.1949 b.1954 b.1881 b.1894 b.1922 b.1928 Eb.1934 b.1967 Eb.1965 Eb.1960 Eb.1933 E+ b.1913 b.1951 b.1979 Eb.1980 E+ b.1983 b.1985 d. 17y E+ b.1912 b.1945 Eb.1941 b.1951 E+ b.1957 Eb.1949 b.1979 E+ b.1982 Eb.1980 E+ b.1979 b.2009 Eb.1969 b.2009 E+ b.2006 b.1974 b.2011 E+ b.2012 E+ b.1984 b.2017 E+ b.2019 b.2019 Ed. 26y [file mmc1.pdf]

Supplemental material: The pedigree.

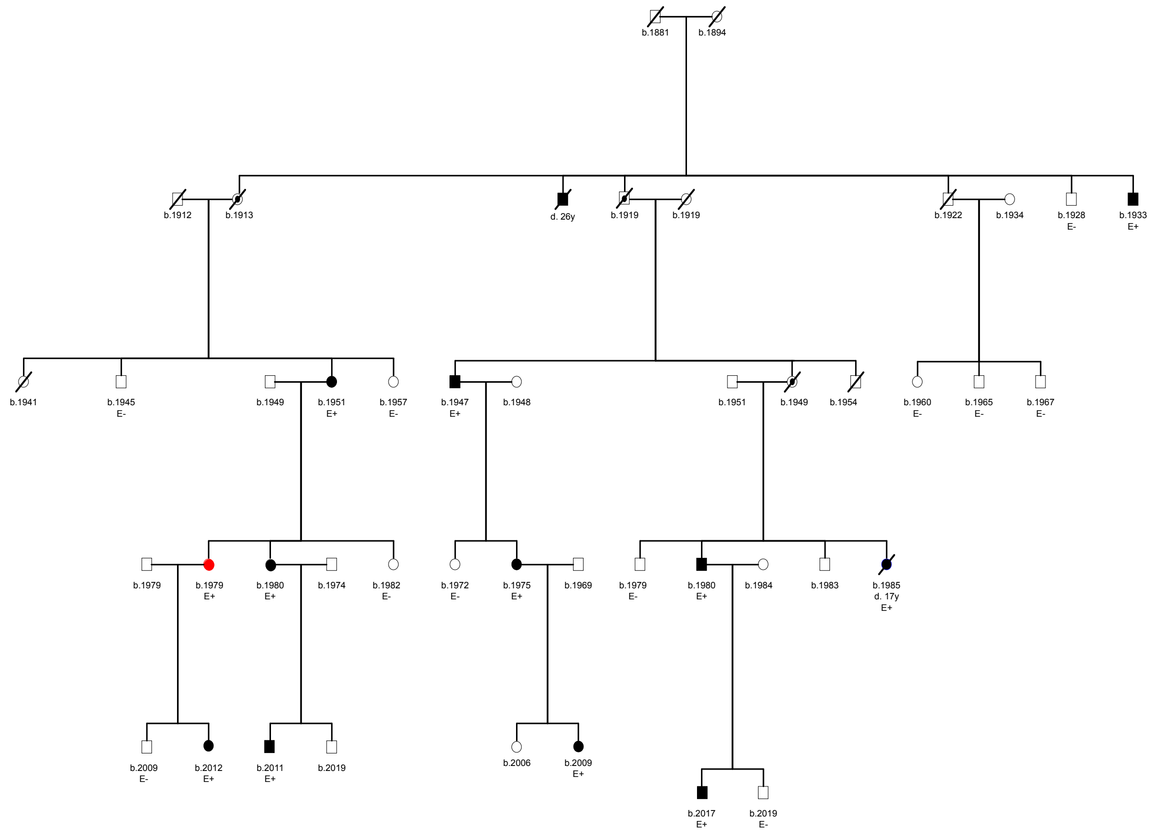

The graph shows the pedigree of the affected family originating from north-western Sweden. Squares: men. Circles: women. Crossed symbols: deceased individuals. E+ and filled symbol depicts individuals positive for the mutation NM_000238.3(KCNH2):c.2593-2A>G. E- depicts individuals, shown to be negative for the mutation. Symbols with central dot depict obligate carriers. The patient is shown as a red symbol.

Two known cases of death at young age (17 and 26 years) can be seen. Additional two cases of sudden death (47 and 54 years) are known to have occurred in the extended family outside the pedigree view.
